# Functional mapping of language networks in the normal brain using a word-association task

**DOI:** 10.4103/0971-3026.69352

**Published:** 2010-08

**Authors:** Shantanu Ghosh, Amrita Basu, Senthil S Kumaran, Subash Khushu

**Affiliations:** Behavioral and Cognitive Science Lab, Department of Humanities and Social Sciences, Indian Institute of Technology Delhi, Hauz Khas, New Delhi 110 016 India; 1 Center for Linguistics, Jawaharlal Nehru University, New Delhi 110 067, India; * Now at Center for Cognitive Science, Jadavpur University, Kolkata 700 032, India; 2 Department of NMR, All India Institute of Medical Sciences, Ansari Nagar, New Delhi 110 029, India; 3 NMR Research Center, Institute of Nuclear Medicine and Allied Sciences, Timarpur, Delhi 110 054, India

**Keywords:** fMRI, language network, lingual gyrus, presurgical planning, word association

## Abstract

**Background:**

Language functions are known to be affected in diverse neurological conditions, including ischemic stroke, traumatic brain injury, and brain tumors. Because language networks are extensive, interpretation of functional data depends on the task completed during evaluation.

**Aim:**

The aim was to map the hemodynamic consequences of word association using functional magnetic resonance imaging (fMRI) in normal human subjects.

**Materials and Methods:**

Ten healthy subjects underwent fMRI scanning with a postlexical access semantic association task vs lexical processing task. The fMRI protocol involved a T2*-weighted gradient-echo echo-planar imaging (GE-EPI) sequence (TR 4523 ms, TE 64 ms, flip angle 90°) with alternate baseline and activation blocks. A total of 78 scans were taken (interscan interval = 3 s) with a total imaging time of 587 s. Functional data were processed in Statistical Parametric Mapping software (SPM2) with 8-mm Gaussian kernel by convolving the blood oxygenation level-dependent (BOLD) signal with an hemodynamic response function estimated by general linear method to generate SPM{*t*} and SPM{*F*} maps.

**Results:**

Single subject analysis of the functional data (FWE-corrected, *P*≤0.001) revealed extensive activation in the frontal lobes, with overlaps among middle frontal gyrus (MFG), superior, and inferior frontal gyri. BOLD activity was also found in the medial frontal gyrus, middle occipital gyrus (MOG), anterior fusiform gyrus, superior and inferior parietal lobules, and to a smaller extent, the thalamus and right anterior cerebellum. Group analysis (FWE-corrected, *P*≤0.001) revealed neural recruitment of bilateral lingual gyri, left MFG, bilateral MOG, left superior occipital gyrus, left fusiform gyrus, bilateral thalami, and right cerebellar areas.

**Conclusions:**

Group data analysis revealed a cerebellar–occipital–fusiform–thalamic network centered around bilateral lingual gyri for word association, thereby indicating how these areas facilitate language comprehension by activating a semantic association network of words processed postlexical access. This finding is important when assessing the extent of cognitive damage and/or recovery and can be used for presurgical planning after optimization.

## Introduction

Functional magnetic resonance imaging (fMRI) may be used for diagnostic purposes to detect language networks of the brain during presurgical planning that include the detection of the dominant hemisphere[1] and localization of critical language and related areas in relation to brain lesions.[2] However, not only are language networks more complex than somatosensory or motor systems, they are far more variable across individuals. The interpretation of complex input from language involves the use of information by the cortical networks, subserving not only the sensory mechanisms but also association cortices and other related networks. This ability not only helps us categorize, organize and integrate linguistic information, it also helps in deriving new concepts from existing ones. The activities subserving such conceptual processing are traditionally posited to rely on the frontal lobe,[3] a large cortical region that interacts with other regions to choose and prepare contextually suitable responses.

This ability of the brain may be affected in several neurological conditions, including ischemic stroke, traumatic brain injury, and brain tumor. In many cases language functions are affected, and often patients need surgical intervention or postsurgical rehabilitative support from speech therapists. The knowledge of normal brain networks for language is crucial for presurgical planning of tumor resection[4] and for post-treatment rehabilitation. Although semantic association between words and its links with memory processes are crucial in language processing,[5] the hemodynamic consequences of the neural mechanisms involved in postlexical access semantic association between words have still not been conclusively determined.

Semantic association between words necessarily requires information to be processed from past memory of words recorded as memory ‘traces,’ which are manipulated using hierarchical networks of syntactic structures and semantic concepts based on word association. Even a relatively simple task such as retrieval of single words is subserved by distributed cortical areas.[6,7] This implies that word-concept processing in the brain is essentially multimodal and is likely distributed over multiple cortical networks. The entire network of language-related circuitry should thus be the focus of investigations during presurgical evaluation and functional recovery. Our choice of baseline and activation was guided by the memory, unification, and control model[5] that theorizes a semantic network to process information extracted from the lexicon to achieve unification. Hence, semantic association between words must be studied separately to ensure that semantic unification networks are covered by the investigations. We therefore investigated the neural substrate of semantic association between already read words, when thematic assignments (information that is semantic in nature) occur at the syntax–semantics interface level or, in other words, when the words have been read already and contextual processing is happening (i.e., postlexical access semantic association).

In this article, we report the results of a blood oxygenation level-dependent (BOLD) fMRI study to demonstrate that apart from the regions that are activated during lexical access (which include bilateral cuneus, left precentral gyrus, left lingual gyrus, right posterior cingulate gyrus, and the right superior and middle temporal gyri [Supplementary data is available online]), there is substantial involvement of the lingual gyri bilaterally during post-lexical access semantic association in a cohort of healthy normal controls. The current investigation has twin purposes: first, it describes the neural substrate of information processing during semantic association of words after they have been accessed from the mental lexicon, and second, to generate data that can be used to compare compromised semantic association-related information processing networks of the brain. The functional imaging protocol described in this study can be used with some modifications to generate functional maps to locate putative language areas in the clinical evaluation of language networks. This may also be helpful for determining the extent of damage in the neural substrates of language among the clinical populations listed above.

## Materials and Methods

### Subjects

Ten healthy multilingual adult volunteers who had learned English as a second language, with at least 13 years of formal education in the English medium (age range 19 – 30 years, mean age 24.3 ± 3.26 years) participated in the experiment. Of these subjects, five were male and five female; all were right handed and had normal vision. The subjects were recruited from among the university students, and screened as per the ethics guidelines for human experimentation laid down by the Helsinki Convention of 1964. All subjects were graduate students with comparable educational backgrounds. The imaging was done at the NMR Research Center, Institute of Nuclear Medicine and Allied Sciences, Delhi, India, after obtaining the requisite clearances from the local Ethics Committee and approval from the Director of the Institute. All subjects gave written informed consent for undergoing the scans.

### Stimuli

The language stimuli for the experiment were prepared from a list of 20 neutral English words (10 nouns and 10 verbs) for the baseline condition, and a list of 20 English word pairs unrelated to the single words for the activation condition. The stimuli were presented using Microsoft PowerPoint^®^ (Microsoft, Redmond, WA) in a randomized block design; thus, there were six task blocks, each consisting of six probe questions in the activation condition (6 × 6 = 36), alternating with seven blocks, each consisting of six baseline stimuli (7 × 6 = 42). The timings of stimulus presentation were synchronized with the data acquisition parameters of the scanner (see below, fMRI data acquisition).

### Task baseline condition

In the baseline condition, words (nouns and verbs) corresponding to the task were presented. The subjects were asked to silently read the word as it was displayed on the liquid crystal display (LCD) screen. The subjects were asked to make noun/non-noun distinction––a lexical decision task. That is, if the word that appeared on the screen was a noun, the participant was asked to record his/her answer by pressing the pneumatic squeeze bulb, using a ‘go/no-go’ response protocol. To appropriately control for movement in conditions where the displayed word was a non-noun, the subject was asked to just clench the fist once, without pressing the pneumatic bulb.

### Activation condition

The activation condition required the subjects to silently read the two words displayed on the screen and indicate whether there was a semantic connection between them. Word pairs selected from nouns and verbs only were randomly presented; both occurred with a probability of ½. In half of the trials, there was a clear-cut connection between the words; in the other half, there was no direct connection. Responses for activation conditions (i.e., postlexical access semantic association/no semantic association, a lexical processing plus semantic association task) were also recorded by pressing a pneumatic squeeze bulb using a ‘go/ no-go’ response protocol. Again, to appropriately control for movement in conditions where the two displayed words were not semantically associated, the subject was asked to just clench the fist once, without pressing the pneumatic bulb. The activation task would thus involve lexical decision and additional semantic processing.

### fMRI data acquisition

The imaging was done using a 1.5-T Magnetom Vision whole-body MR scanner (Siemens AG, Erlangen, Germany). High-resolution T1-weighted anatomical scans, with 39 transverse slices to cover the whole brain (scan repeat time, TR 1833 ms, echo time, TE 15.0 ms), were obtained for all subjects after 3D shimming of the regions of interest (ROIs). Thereafter, BOLD functional images, history-matched to the anatomical scan, were acquired continuously for 587 s, using a T2*-weighted gradient-echo echo-planar imaging (GE-EPI) sequence with a circularly polarized head coil (TR 4523 ms, TE 64 ms, flip angle 90°, slice thickness 3 mm, slice shift 5.8 mm, a 230 mm field of view, matrix size 64 × 128, and spatial resolution 2.8 × 2.8 mm^2^). Head movement was restricted using foam padding. A total of 78 scans were obtained in a blocked design with alternating baseline and activation phases, with a baseline phase at the end; the interscan interval was 3 s. During scanning, the language stimuli were administered using MR-compatible binocular LCD eyepieces (SV-2200, Avotec, Jensen Beach, FL) from a laptop (Siemens Nixdorf, Munich, Germany). The laptop was also used for synchronization of visual language stimuli presentation with the scanning. The visual stimuli were routed through SVGA-to-PAL-M/N signal converter (UltiMate 2000AX, Grandtec, Taiwan) via a fiberoptic cable. The responses of the subjects were monitored using a pneumatic squeeze bulb.

### fMRI data analysis

Data analysis was done using statistical parametric mapping (SPM2, Wellcome Department of Cognitive Neurology, London, UK, www.fil.ion.ucl.ac.uk/spm/) implemented in MATLAB (Matlab 6.5, MathWorks, Natick, MA) in Windows XP^®^ (Microsoft) environment. The fMRI data from each participant were slice acquisition-corrected, motion-corrected, and coregistered to the coplanar anatomical image from each participant and represented in a stereotaxic atlas,[8] a standard brain-space coordinate system for anatomical reference. The first six measurements (baseline) were not considered for analysis to compensate for T1 saturation effects. The T1 images were normalized to the standard SPM template with the transformation matrix applied to the coregistered functional images. For each scan in the present experiment, the hemodynamic response function (HRF) was estimated by correlating the activity of each voxel to a reference function obtained by convolving the square wave describing the task alternation. These correlation values were then normalized to create a functional image SPM{*t*} for each individual scan for each subject. The normalized functional images, spatially smoothed with a Gaussian filter (8-mm full-width at halfmaximum kernel) and interpolated to 3-mm isotropic voxels, were entered into a regression function for a random-effects analysis using a general linear model[9] to generate a functional image SPM{*F*} for within-group effects at *P*≤0.001. The *P* values were corrected for family-wise errors (FWE) for multiple comparisons.

The ROIs obtained from the MNI coordinates using a nonlinear transform mni2tal (http://imaging.mrc-cbu.cam.ac.uk/imaging/MniTalairach) and Talairach Client 2.4.2 (http://www.nitrc.org/projects/tal-daemon/) were indicated using the Brodmann areas (the coordinates referring to either the geometric center of mass of the voxel, showing maximum activation or the center of the voxel, showing maximum BOLD contrast). Using a data-derived estimate of HRF, a delay of 2 to 3 s was assumed during the time course of the initiation of the maximal activation (increase in BOLD response with time), before returning to baseline 8 s later.[10] On two occasions, the scans had to be repeated because the motion parameters were more than 1.5 mm (translational) and 1° (rotational). A brain area was included in Table 1 if the cluster count was more than five and was at least two voxels away from the nearest maximum.

## Results

### Single subject analysis

First-level analysis of the functional data (corrected, *P*≤0.001) revealed that there is extensive activation in the frontal lobe in all 10 subjects during execution of the postlexical access semantic association task, with overlaps among middle frontal gyrus (MFG) and superior frontal gyrus (SFG) and/ or between MFG and inferior frontal gyrus (IFG). BOLD activity was also reported from the right medial frontal gyrus and in the middle occipital gyrus for all subjects, while no pattern was evident in the inferior temporal gyrus, for which activity was found in two subjects only. The anterior fusiform gyrus, superior and inferior parietal lobules, thalamus, and right anterior cerebellum were also implicated in eight of the ten subjects.

### ROI main-effects analysis

**Figure 1 (A-C):**
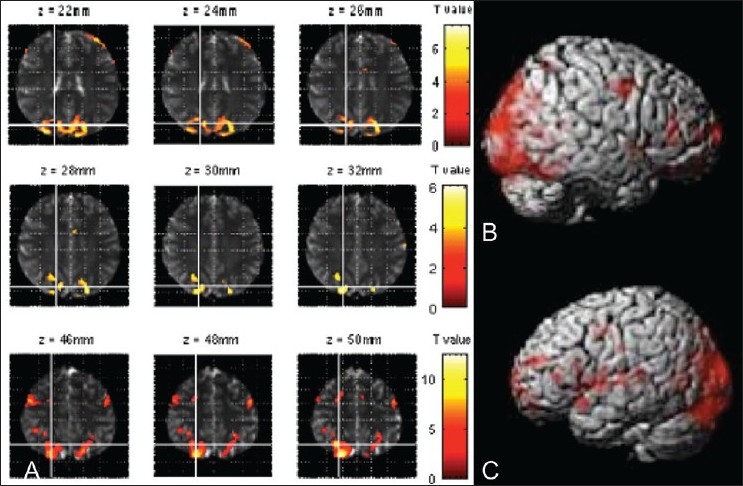
MRI images (A) show the cortical activation pattern for the whole sample for postlexical access semantic association task. Functional scans were done in a 1.5-T whole-body scanner and overlaid on an SPM anatomical template. Surface rendering of cortical activation for word concept association for all subjects for the right (B) and left (C) hemispheres (FWE-corrected, *P*≤0.001).

Group analysis (FWE-corrected, *P*≤0.001) revealed a pattern of neural recruitment that is centered around the lingual gyri bilaterally, middle occipital and fusiform gyri, and the thalamic region. The Montreal Neurological Institute coordinates of the significant activation sites found in the group analysis of the sample is depicted in Table 1 (Figure 1A depicts the functional activations overlaid on anatomical template image for all subjects pooled together, and Figure 1B and C illustrate cortical rendering of the same data in the right and left hemispheres). We found that the lingual gyri bilaterally are activated along with the left MFG, the middle occipital gyri bilaterally, left superior occipital gyrus, left fusiform gyrus, both thalami, and the right cerebellar areas to effect postlexical access semantic association.

**Table 1:** Montreal Neurological Institute (MNI) coordinates of cortical areas active for postlexical access semantic association

Brain region	z-score	MNI coordinates (x, y, z)	Cluster count
Bilateral			
Lingual gyrus/cuneus	5.07	-16, -78, -8	2461
	4.99	22, -74, 10	
Left hemisphere			
Superior occipital gyrus	4.07	-30, -74,20	25
Middle occipital gyrus	3. 79 3.68	-36, -18, -2 to -51, -66, -5	55
Fusiform gyrus	4.09	-24, -51, -11	11
Middle frontal gyrus	3.66	-42, 3, 53	10
Precuneus	3.39	-24, -50, 49	8
Posterior cingulate gyrus	3.21	-10, -68, 6	4
Right hemisphere			
Middle occipital gyrus	3.62	40, -78, 6	31
Cuneus	3.34	10, -86, 37	20
Posterior cingulate gyrus	3.42	16, -56, 6	9
Superior parietal lobule	3.43	28, -53, -58	8
Fusiform gyrus	3.35	26, -82, -16	3
Subcortical and other areas			
L Thalamus/Sub-lobar	3.61 3.19	0, -14, 12 to -8, -9, 12	8
L Sublobar/lentiform nucleus	3.88	-28, -18, -2	5
R Cerebellum/declive/culmen	3.49	4, -77, -18	20
R Thalamus/brainstem	3.26	2, -31, 2	7

## Discussion

Our results indicate that a cerebellar-occipital-thalamic network is activated when the semantic relatedness of words are assessed by the brain. In addition, the high cluster count from the left lingual gyrus provides evidence that indicate widespread processing of associated semantic information. Semantic association tasks such as the one used in the current study are, besides being a measure of language fluency, an indication of the extent of executive function and working memory. These reported areas have been closely linked in higher-order cognitive functions such as phonological-semantic connections (right cerebellum) and lexical code connections (lingual gyrus).[11] These areas could thus be playing a role in the transfer of semantic information to a more permanent storage format in the cortical association areas. Hence, the lingual gyrus activity may be linked to content-specific retrieval ‘orientation’[12] relevant to recall, association, and integration of wordrelated information.

In our findings, the maintenance of semantic information in working memory appears to recruit a network that depends critically upon interconnected brain regions that include predominantly the occipital and the parietal cortices, as well as the left middle frontal lobe. This is in partial consonance with earlier reports of the prefrontal cortex, functioning as the central executive for integration of information.[3] Activation of the left fusiform gyrus in our study may be linked to context integration, possibly due to an encoding-related activation during subsequent retrieval for recognition.[13] For remembered words too, significant activation has been reported to occur in the fusiform gyri bilaterally, possibly due to memory retrieval.[14] The activation *vs* baseline contrast revealed in our study isolates the neural substrate for postlexical access semantic association of words. The subtractive nature of the fMRI design contrast would likely have eliminated or at least attenuated to a large extent any visual or lexical processing likely to contaminate the word-level semantic association-related BOLD activity by the nonassociationrelated BOLD signals. The reported activity in the present experiment may thus be attributed to online semantic association of word-concept information, already stored as abstract representations in our mental lexicon. The current study characterizes the neural substrate relevant to the cognitive mechanisms that are likely implemented by word semantics-dependent information processing, leading to concept formation contingent on word-association of already stored lexical concepts. We propose that the neural mechanisms of memory needed for lexical informationbased semantic association must therefore be capable of multiple forms of encoding to establish unification of semantic processing. This is distinct from episodic memory processes that also allow neural ensembles to switch between functional states. Arguably, the brain uses this cerebellar-occipital-thalamic network to activate novel word information in phonologically-encoded lexical units, constantly refreshing the buffer as new word concepts form. The cross-talk between the cerebellar-occipital-thalamic cortical areas is thus found to be crucial for word-concept association, a phenomenon thought to be a prerequisite for language comprehension.

When complex neural computations synchronize these subsystems, thus binding them into a higher order system, the semantic content of the word concepts are linked with grammatical encoding to generate language comprehension. The process therefore requires substantial working memory resources. Because the neural correlates of the verbal component of working memory have already been dissociated into the left supramarginal gyrus as the locus of the phonological store and the subvocal rehearsal system traced to the Broca’s area,[15] it is expected that cooperating networks involving these cortical areas would also be critically involved in comprehension and concept formation. Thus, in order to determine how word concept associations are formed, our examination of the neural mechanisms specific to these two processes (postlexical access semantic association and memory retrieval) has revealed that an additional store must be present for online semantic processing localized in the lingual gyrus bilaterally, separate from lexical and functional semantic memories. However, the small variabilities in our subjectwise data may be due to the fact that the semantic status of real propositional constructions is likely influenced by word-association judgments and attentional modulations. The source of such semantic-conceptual processing has been placed at the lingual/fusiform gyri bilaterally using brain electric dipole analysis.[16] It may be reasoned that since meaning is relevant to the semantic association task at hand, participants automatically divert some of their attention to the meanings present in the words. Our results are consistent with previous results that reveal interactions between lexical and perceptual processes in word analyses.[3] The complexity of these interactions probably may be one of the reasons why an unambiguous correlation has not emerged about the relation between semantic and word concept association in the left frontal cortex. However, our experiment has uncovered the possible existence of a separate postlexical semantic association substrate, revealing the involvement of a possible cerebellar-occipitalfusiform- thalamic information-processing network during word concept association.

We have shown here that fMRI can precisely map the semantic association areas of the normal brain mediated by information from the mental lexicon, and hence would be of great help in preoperative decision-making for patients who have brain tumors or patients who require surgery for refractory epilepsy or traumatic brain injury involving the lingual gyrus bilaterally. Whether it is before a patient has surgery for removing a tumor, or for evaluating the extent of damage/recovery either intraoperatively or postoperatively, the functional mapping protocol for word association delineated here could be administered with ease. In both cases, fMRI is a promising noninvasive technique and is strongly indicated in patients whose lesions are situated adjacent to or within critical language areas. In this study, we employed a blocked design for language fMRI in ten healthy controls and used a task involving semantic association between words. Our results suggest that the proposed blocked design for localizing language functions may generate sensitive task-related language maps, and has the potential for use in presurgical decision-making and postsurgical evaluation.

The results of this study should be interpreted with caution for several reasons. The study was conducted on a small sample and hence needs to be supported by additional studies. A mixed-effect age- and sex-matched study with patients with different neuropathies would also need to be done to determine the effectiveness of the protocol for routine clinical use. The general linear model used in the current study needs to be compared with newer, more robust, dynamic causal models. Finally, a comparison with event-related paradigms to see which is more likely to detect the semantic word-association network needs to be carried out. To summarize, more studies involving both patients and healthy controls and using multiple methodologies need to be carried out to further investigate and optimize this approach for presurgical planning.
